# Bilateral macular hole formation secondary to sclopetaria caused by shockwaves transmitted by a posterior vector: case report

**DOI:** 10.1186/1471-2415-10-6

**Published:** 2010-03-19

**Authors:** Nancy Kunjukunju, Alicia Navarro, Scott Oliver, Jeff Olson, Chirag Patel, Gerardo Garcia, Naresh Mandava, Hugo Quiroz-Mercado

**Affiliations:** 1 Retina and Vitreous Center of Southern Oregon, Ashland, Oregon, USA; 2 Asociación para Evitar la Ceguera en México, Mexico City, Mexico; 3 Rocky Mountain Lions Eye Institute, University of Colorado Hospital, Aurora, Colorado, USA

## Abstract

**Background:**

Sclopetaria is a rare ophthalmic finding in trauma

**Case Presentation:**

This is a report of a patient who developed macular holes from sclopetaria induced by indirect trauma. A 22-year-old male, suffered a gunshot wound that passed behind his eyes, resulting in bilateral macular hole formation

**Conclusion:**

To our knowledge, this is the first reported case in which trauma posterior to the globes caused bilateral macular hole formation

## Background

The mechanism underlying idiopathic macular hole formation has been well elucidated by optical coherence tomography (OCT), particularly with regard to the relationship between the posterior vitreous cortex and the macular area [1,2]. Tangential and anterior-posterior forces at the fovea have been implicated in idiopathic macular hole formation [3]. There is no longer a great deal of speculation or controversy regarding perifoveal vitreous traction as a mechanism for idiopathic macular hole formation. In traumatic macular holes however, such is not the case. One commonly held theory is that blunt trauma directly alters the anterior-posterior diameter of the globe in such a fashion as to induce stress on the retina at significant areas of vitreous attachment [4]. Most theories regarding traumatic macular holes involve direct blunt contact with the globe. We present an interesting case of a patient who sustained bilateral macular hole formation indirectly, with no evidence of direct trauma to the globe.

## Case Presentation

**Figure 1:**
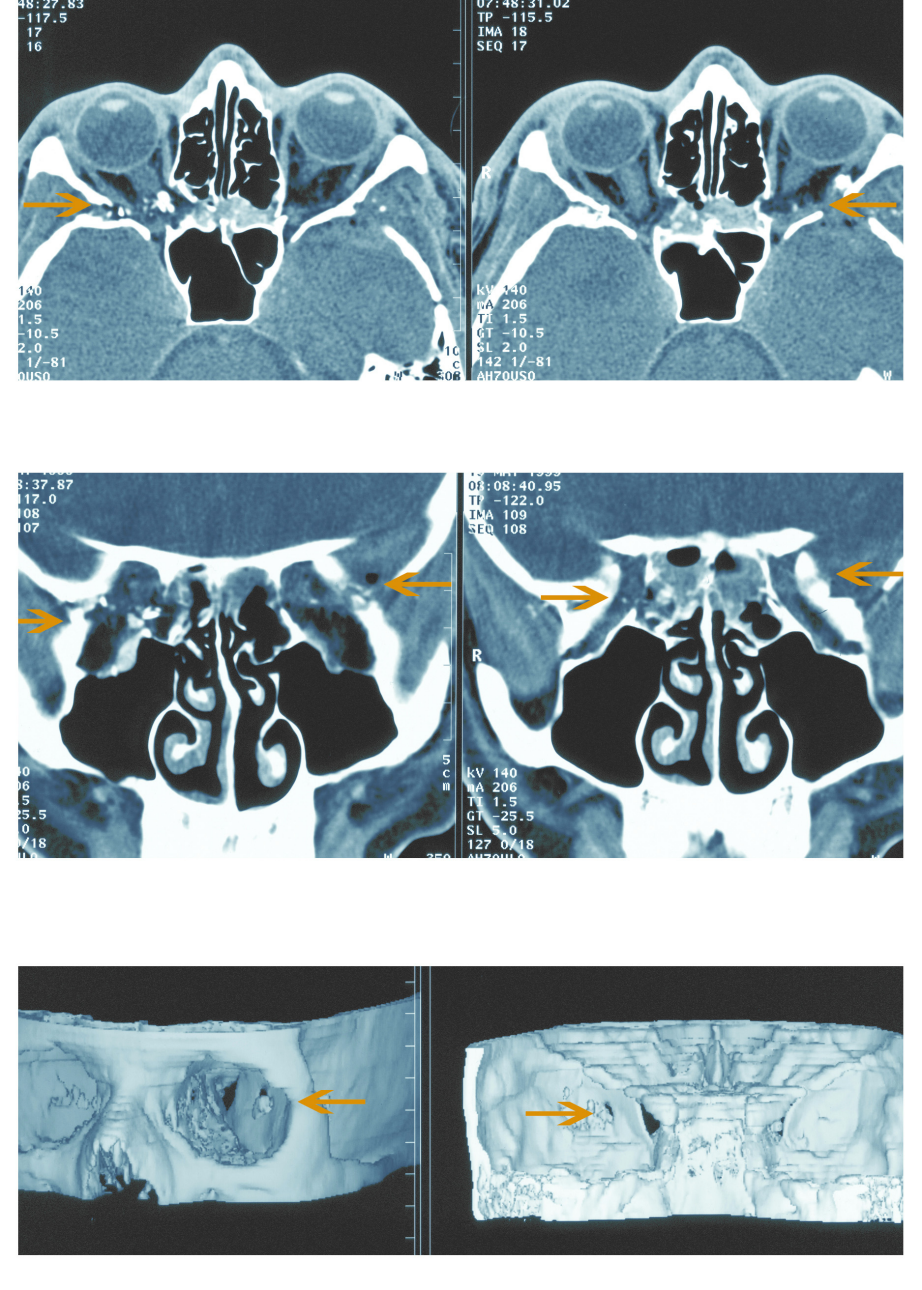
**Entrance and exit wounds (see arrows) revealed by computerized tomography (CT) scans (axial and coronal views) in conjunction with three-dimensional (3D) orbital reconstruction (anterior and posterior views)**.

**Figure 2:**
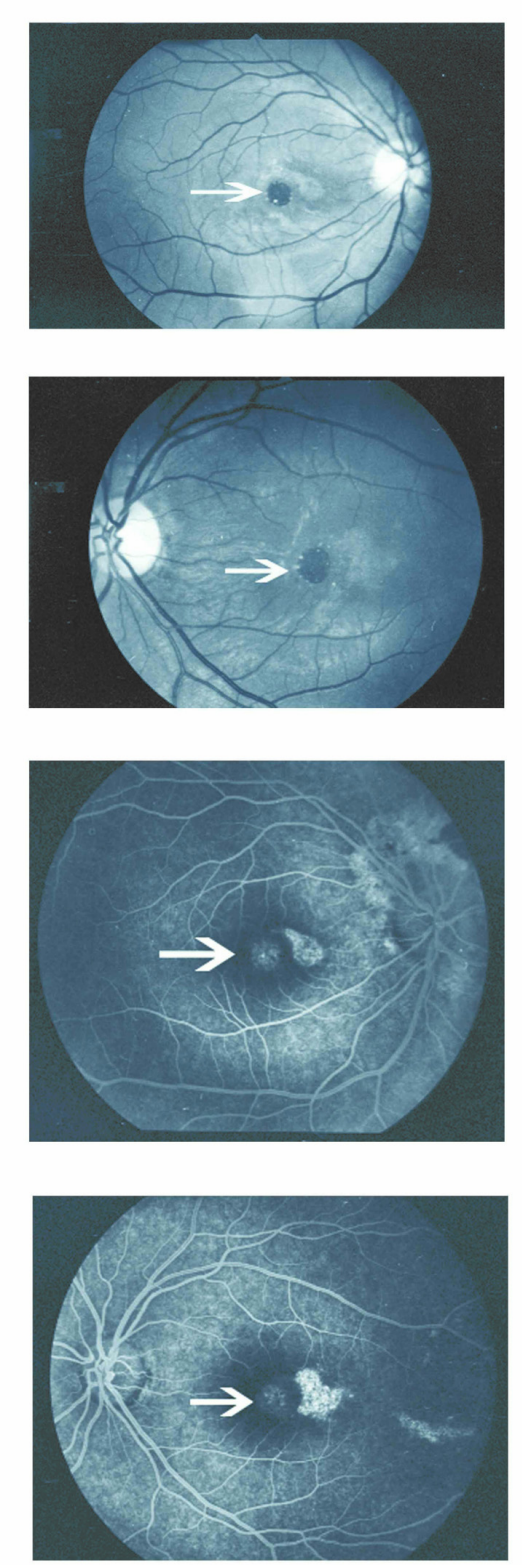
**Red free and late fluorescein images (OU), with late fluorescein images demonstrating window defect at the site of the macular hole**.

A 22-year-old male patient with no prior medical or ocular history was referred for evaluation. The patient suffered a gunshot wound to the head. On examination the vision was 20/60 in both eyes; his intraocular pressures were 18 and he had normal extra-ocular movements and pupils. The anterior segment appeared benign. The optic nerves were normal in color and there was no evidence of swelling or elevation; there were some mild areas of peripapillary commotio in the right eye. There were pigmentary changes surrounding the fovea of both eyes. The peripheral retina was normal; there was no evidence of breaks or tears. The patient underwent a computerized axial tomography (CT) scan, and had a three-dimensional (3D) reconstruction of his injury (Figure 1). Both CT scan and 3D imaging confirmed that there was no direct injury to the globe. The bullet passed through the posterior orbit without hitting the globe or optic nerves. Over the next few days, he developed a macular hole in both eyes (Figure 2). The right eye also developed a proliferation of fibrous tissue temporal to the fovea. He had surgery for the hole in his left eye 4-5 months after the incident. Clinically, there was no evidence of posterior vitreous detachment. He underwent pars plana vitrectomy, with surgically induced posterior vitreous detachment, peeling of the internal limiting membrane and air-fluid-gas exchange. The hole initially closed, but reopened 1 month later with a final visual acuity of 20/200. The patient elected to have no further surgery.

## Conclusions

There are a number of theories regarding the underlying cause of traumatic macular hole formation. It has been suggested that the direct force of the impact transmitted to the macula causes foveal rupture [5]. Cyst formation and rupture of the cyst has also been implicated [5]. Although sudden vitreous separation has also been thought to be the cause of traumatic macular hole formation, other cases have documented an attached posterior vitreous at the time of vitrectomy [6]. Johnson et al. has hypothesized that there is a "trampoline-like movement of the posterior pole," that causes tractional forces on the retina when there is sudden compression of the globe in blunt trauma [6]. Immediate visual loss is believed to be secondary to foveal detachment, while delayed visual loss in other patients may be due to accumulative cystic changes and photoreceptor loss [5,7]. The aforementioned theories characterize macular hole formation secondary to trauma directly affecting the globe.

Our patient's eyes were indirectly affected by trauma and the previously noted theories are not applicable. Rather it is our hypothesis that the etiology of our patient's macular holes may have been caused by a mechanism similar to that of chorioretinal ruptures caused by nonpenetrating injury. Sclopetaria or chorioretinal rupture is a rare manifestation of nonpenetrating ocular trauma. Essentially, a high-velocity object triggers a force or "shock-wave" that deforms the globe and is transmitted to the posterior vitreous, retina, choroid and sclera [8]. The shock wave causes simultaneous damage to the retina and choroid; these tissues are disrupted revealing bare sclera. The mechanical disruption and tissue retraction causes a full-thickness chorioretinal defect and visual loss. It has been postulated that shock waves transmitted in an anterior-posterior direction, as in the case of a Nd:Yag laser peripheral iridotomy, may induce a macular hole. In this report they hypothesize that the laser generated shock waves, due to plasma formation and photo-disruption, eventually led to vitreous traction and macular hole formation [9]. Theories have also been postulated regarding an acoustic shock wave as a possible mechanism of retinal damage [10].

Sclopeteria is caused by shock waves. Sclopetaria and macular hole formation is a rare association following trauma [11]; indeed our case represents an unusual presentation of bilateral traumatic macular hole formation resulting from shock waves transmitted from behind the globe in a posterior-anterior direction. Our patient suffered a dehiscence of retinal tissue at the fovea as a result of these shockwaves, as in sclopetaria, which induced bilateral macular hole formation.

Although there are cases in which traumatic macular holes may close spontaneously [12] and others cases in which surgery has been successfully performed to close them [6], our patient's visual outcome was poor. As in the case of laser injury [13] in which there can be permanent damage to the photoreceptor cells, our patient's photoreceptors may have been affected. Spontaneous resolution of traumatic holes has been observed in cases in which there is vitreous adhesion at the edges of the hole and is believed to be secondary to a complete posterior vitreous detachment [14]. Our patient was observed for a period of time, as he was young and there was vitreous adhesion [15]. However, he eventually underwent surgery. Although his macular hole initially closed, it opened later. The mechanism for the reopening is unclear, particularly as there seemed to be no other underlying reason for failure of the hole to close: there was no evidence of further traction nor did the patient undergo cataract surgery [16,17]. It is possible that a stable glial plug failed to form [18]. In any case, our patient opted not to have further surgery.

## Competing interests

The authors declare that they have no competing interests.

## Authors' contributions

NK participated in writing and editing the manuscript. NA examined and evaluated the patient. SO participated in editing the manuscript. JO participated in editing the manuscript. CP participated in editing the manuscript. GG examined and evaluated the patient. NM participated in writing and editing the manuscript. HQ examined and evaluated the patient, in addition to evaluating the manuscript. All authors read and approved the final manuscript.

## Pre-publication history

The pre-publication history for this paper can be accessed here:

http://www.biomedcentral.com/1471-2415/10/6/prepub
